# Levels of Germination Proteins in *Bacillus subtilis* Dormant, Superdormant, and Germinating Spores

**DOI:** 10.1371/journal.pone.0095781

**Published:** 2014-04-21

**Authors:** Yan Chen, W. Keith Ray, Richard F. Helm, Stephen B. Melville, David L. Popham

**Affiliations:** 1 Department of Biological Sciences, Virginia Tech, Blacksburg, Virginia, United States of America; 2 Department of Biochemistry, Virginia Tech, Blacksburg, Virginia, United States of America; Universidad Andres Bello, Chile

## Abstract

Bacterial endospores exhibit extreme resistance to most conditions that rapidly kill other life forms, remaining viable in this dormant state for centuries or longer. While the majority of *Bacillus subtilis* dormant spores germinate rapidly in response to nutrient germinants, a small subpopulation termed superdormant spores are resistant to germination, potentially evading antibiotic and/or decontamination strategies. In an effort to better understand the underlying mechanisms of superdormancy, membrane-associated proteins were isolated from populations of *B. subtilis* dormant, superdormant, and germinated spores, and the relative abundance of 11 germination-related proteins was determined using multiple-reaction-monitoring liquid chromatography-mass spectrometry assays. GerAC, GerKC, and GerD were significantly less abundant in the membrane fractions obtained from superdormant spores than those derived from dormant spores. The amounts of YpeB, GerD, PrkC, GerAC, and GerKC recovered in membrane fractions decreased significantly during germination. Lipoproteins, as a protein class, decreased during spore germination, while YpeB appeared to be specifically degraded. Some protein abundance differences between membrane fractions of dormant and superdormant spores resemble protein changes that take place during germination, suggesting that the superdormant spore isolation procedure may have resulted in early, non-committal germination-associated changes. In addition to low levels of germinant receptor proteins, a deficiency in the GerD lipoprotein may contribute to heterogeneity of spore germination rates. Understanding the reasons for superdormancy may allow for better spore decontamination procedures.

## Introduction

Bacterial endospores are metabolically dormant and resistant to a variety of anti-microbial treatments due to their protective structures and dehydrated spore core [1], [2]. These spores can survive for decades in the absence of nutrients. However, they are able to return to a metabolically active state through a series of events termed spore germination. Once spores lose many of their resistance properties during germination, they can then be easily eliminated by routine decontamination methods [3]. Since the spores of *Bacillus* and *Clostridium* species cause food spoilage and are infectious agents in several human diseases [4], the development of methods or reagents that stimulate highly efficient germination across a spore population could greatly simplify decontamination efforts and reduce morbidity and mortality.

Procedures used for triggering spore germination do not achieve 100% efficiency due to heterogeneity in germination rate within spore populations. Studies of single germinating *B. cereus* and *Clostridium* spores indicated that the spore germination heterogeneity results from the variation in time of initiation of rapid Ca^2+^-dipicolinic acid (DPA) release (T_lag_) [5], [6]. Subpopulations of *B. subtilis* spores termed superdormant spores can be isolated following multiple rounds of germination with saturating nutrient germinant levels [7]. These superdormant spores exhibit extremely poor germination response to the germinant used for isolation, but will germinate to varying degrees when triggered with germinants that utilize other germinant receptors [7], [8]. Individual germinating superdormant spores exhibit longer times for initiation of rapid Ca^2+^-DPA release relative to initial dormant spore populations [8]. However, once rapid Ca^2+^-DPA release is initiated, the rate of release is similar for all spores. Thus one may hypothesize that the state of superdormancy is related to processes occurring prior to Ca^2+^-DPA release.

Four groups of proteins have been implicated to be involved in the early steps of germination: 1) germinant receptors; 2) DPA channel proteins; 3) germination-specific lytic enzymes (GSLEs) and their partner proteins; and 4) lipoproteins potentially involved in transducing germinant-binding signals [3], [9]. In *B. subtilis*, three major germinant receptors (GRs) have been characterized: GerA, GerB, and GerK [10]–[12]. Each GR is comprised of at least A, B, and C subunits (some receptors have D subunits encoded within or associated with the receptor operon [13]) and is localized to the spore inner membrane. The A and B subunits are believed to be integral membrane proteins with multiple transmembrane domains. The C subunits are putative lipoproteins based on their N-terminal signal peptides and on the effect of a *gerF* mutation, which eliminates the only protein diacylglycerol transferase in this species, on their function [14].

Previous genetic studies of these GRs illustrated their germinant specificity. GerA alone responds to L-alanine or L-valine, while GerB and GerK are required for germination with a mixture of L-asparagine, D-glucose, D-fructose, and potassium ions (AGFK) [12], [15]. The binding of nutrient germinants to their cognate GR or GRs initiates irreversible germination activation [3], and via an unclear pathway, results in the opening of DPA channels and the rapid release of this abundant spore solute. Proteins encoded by the *spoVA* operon are involved in DPA uptake during sporulation as well as release during spore germination. Since SpoVA proteins are transcribed exclusively in the developing forespore and some appear to be integral membrane proteins, they are most likely localized to the inner spore membrane [16]–[18]. The PrkC protein has been identified as an alternate class of germinant receptor that recognized the presence of peptidoglycan fragments in the medium [19].

Complete germination requires that the thick layer of spore cortex peptidoglycan be degraded by GSLEs [20]–[22]. SleB is a key GSLE [21], and some evidence indicates that it and a co-expressed protein involved in SleB stabilization, YpeB, are localized to the inner spore membrane in the dormant spore [23]. Spores with a *gerD* deletion mutation had a dramatically slower response to nutrient germinants utilizing any of the Ger receptors [24]. Lipoproteins involved in germination, including GerAC, GerBC, GerKC and GerD, are believed to be anchored in the spore inner membrane by a covalently attached lipid [14]. Spores of a *B. subtilis gerF* null mutant also lacked both the GerAC and GerD proteins [25]. This mutant exhibited a significant defect in germination with a greater effect on germination triggered through the GerA receptor relative to responses via the GerB and GerK receptors [14], [25].

Several studies have indicated that the abundance of germination-associated proteins can impact the rate of spore germination. Overexpression of the GerA receptor significantly increased the germination rate triggered by its corresponded germinants but did not affect GerB and GerK abundance or germination function [26]. In contrast, overexpression of SpoVA proteins increased germination rates triggered through any germinant receptor [27]. It is hypothesized, based upon quantitative Western blot analyses, that a significant reduction in the amount of a Ger receptor could be the reason for spore superdormancy [28].

In an effort to provide additional insight into the mechanisms of germination, we developed a multiple-reaction monitoring (MRM) mass spectrometry assay [29] to quantify 11 germination proteins believed to be associated with the spore inner membrane. MRM assays are based upon the analyses of peptides specific to the target protein (proteotypic peptides), which become surrogates for protein abundance. The method has high specificity and sensitivity for target protein quantification, and permits reproducible analyses of multiple samples. MRM analyses were performed on membrane preparations obtained from dormant, rapidly germinating, and superdormant spore samples. The results of these analyses indicate that the GerD lipoprotein level can contribute to the heterogeneity of spore germination rate and superdormancy.

## Materials and Methods

### Spore Sample Preparation

The *B. subtilis* strain used was PS832, a prototrophic laboratory derivative of strain 168. Spores were prepared on 2xSG [30] agar plates without antibiotics. Spores were harvested after 72 h incubation at 37°C and purified by water washing and centrifugation through a 50% sodium diatrizoate (Sigma) layer as described [31]. All spores used in this work were 99% free of vegetative cells and were stored in deionized water at 4°C until analysis.

A 10-ml suspension of dormant spores at an optical density at 600 nm (OD_600_) of 20 in water were heat-activated at 75°C for 30 min and cooled on ice for at least 10 min. The spores were then germinated at 37°C and at an OD_600_ of 2 with 10 mM L-valine in 25 mM Tris-HCl buffer (pH 7.4). The germination of spores was terminated after the OD_600_ dropped to 50% of the initial value. Germinated spores were collected by centrifugation at 12,000x*g* for 5 min at 4°C, quickly washed with cold deionized water, centrifuged again, and frozen at −80°C. Examination by phase-contrast microscopy indicated that >95% of the spores in these preparations had germinated.

Superdormant spores were isolated and characterized as described previously [7]. Briefly, dormant spores at OD_600_ of 1 were germinated as described above for 2 h and collected by centrifugation. The pellet was washed with deionized water, suspended in 20% w/v sodium diatrizoate, and centrifuged through a 50% w/v sodium diatrizoate solution (13,000×*g* for 45 min) to separate dormant spores from germinated spores. The dormant spore pellets were collected and washed thoroughly with deionized water. These dormant spores were subjected to another 2 h round of germination and were separated by density gradient centrifugation again. The final superdormant spore pellet was washed thoroughly with deionized water and stored at 4°C.

### Superdormant Spore Characterization

For phenotypic studies, isolated superdormant spores as well as initial dormant spores were germinated with nutrient germinants: 10 mM L-Valine or AGFK (13 mM L-asparagine, 13 mM D-glucose, 13 mM D-fructose, 13 mM KPO_4_ [pH 7.4]); or the non-nutrient germinant 60 mM Ca^2+^-DPA [pH 7.4]. Prior to nutrient-triggered germination, spores were heat-activated in water at 75°C for 30 min and then briefly cooled on ice. Germination was initiated by diluting spores to an OD_600_ of 0.2 in germination solutions and incubating at 37°C. Germination was monitored as the change in OD_600_ over time. Spores used for Ca^2+^-DPA germination were not heat-activated and the germination was at 30°C. To assess Ca^2+^-DPA germination, 100 spores were examined by phase-contrast microscopy at several incubation time points.

### Preparation of Spore Membrane Fractions

Spore membrane fractions were prepared by a modification of previously described methods [32]–[34]. Dormant, germinated, and superdormant spores prepared as described above were lyophilized. The dry spores (∼19 mg for germinated spores and ∼24 mg for dormant and superdormant spores) were pulverized with 100 mg of glass beads in a dental amalgamator (Wig-L-Bug) at 4,600 rpm for pulses of 30 s each, with 30 s pauses on ice between pulses. Spore disruption was monitored by suspending a small sample of spore material in H_2_O and observing under phase-contrast microscopy. Once >80% of spores were disrupted, the dry powder was suspended in 0.5 ml of 4°C extraction buffer (10 mM Tris-HCl [pH 7.4], 1 mM EDTA, 2 mg/ml RNase A, 2 mg/ml DNase I, 1 mM phenylmethylsulfonyl fluoride (PMSF)). The suspension was centrifuged (6,000×*g*, 10 min, 4°C) and the resultant supernatant was centrifuged again (13,000×*g*, 10 min, 4°C) to remove insoluble material. The remaining supernatant was subjected to ultracentrifugation (100,000×*g*, 60 min, 4°C). The resulting supernatant was considered the spore core soluble fraction and was stored at −80°C. The resulting pellet, designated the crude spore membrane fraction, was homogenized in 1 ml high salt buffer (20 mM Tris-HCl [pH 7.5], 10 mM EDTA, 1 M NaCl, 1 mM PMSF) and was gently shaken for 30 min at 4°C. The homogenate was subjected to ultracentrifugation again as described above. The remaining pellet was homogenized in 1 ml alkaline buffer (100 mM Na_2_CO_3_-HCl [pH 11], 10 mM EDTA, 100 mM NaCl, 1 mM PMSF) and was again subjected to ultracentrifugation. After a final wash with 1 ml TE buffer (10 mM Tris-HCl [pH 7.4], 1 mM EDTA, 1 mM PMSF), the resulting pellet was homogenized in 200 µl TE buffer, flash frozen, and stored at −80°C until analysis. The protein concentration was determined by acid hydrolysis and amino acid analysis [35] with comparison to a standard set of amino acids (Sigma).

### Protein Digestion

Proteins in spore membrane fractions (70 µg) were precipitated with 1 mL of acetone −20°C overnight and collected by centrifugation for 20 min at 12,000 g. Protein was resuspended in 250 µl of freshly-prepared 8 M urea, 20 mM Tris-HCl, pH 8.0 to give a final protein concentration of 1 mg/ml. Proteins were denatured by the addition of 27.8 µl of freshly-prepared 45 mM dithiothreitol, 20 mM Tris-HCl, pH 8.0, and incubation for 1 h at 37°C. Free cysteines were alkylated by the addition of 30.9 µl of freshly-prepared 100 mM iodoacetamide, 20 mM Tris-HCl, pH 8.0, incubation at room temperature in the dark for 30 min. Unreacted iodoacetamide was inactivated by the addition of 102.9 µl of freshly-prepared 45 mM dithiothreitol, 20 mM Tris-HCl, pH 8.0. Proteins were digested by the addition of 1.03 ml of 20 mM Tris-HCl, pH 8.0, and 5 µg trypsin in 10 µl 50 mM acetic acid followed by incubation overnight at 37°C with shaking. Trifluoroacetic acid was added to a final concentration of 0.25% and formic acid was added to a final concentration of 1%. The pH was measured and additional formic acid was added until the pH was at or below 3. Conditioning of 0.1 ml OMIX C18 solid phase extraction cartridges used 0.2 ml methanol, followed by 0.2 ml 50% acetonitrile, 0.1% TFA and finally 0.2 ml 2% acetonitrile, 0.1% TFA. A protein sample was applied to the cartridge, which was then washed three times with 0.2 ml 2% acetonitrile, 0.1% TFA. Peptides were eluted with 0.2 ml 75% acetonitrile, dried, and resuspended in 0.02 ml solvent A (2∶98 acetonitrile∶water containing 0.1% formic acid).

### Liquid Chromatography and Mass Spectrometry

**Figure 1:**
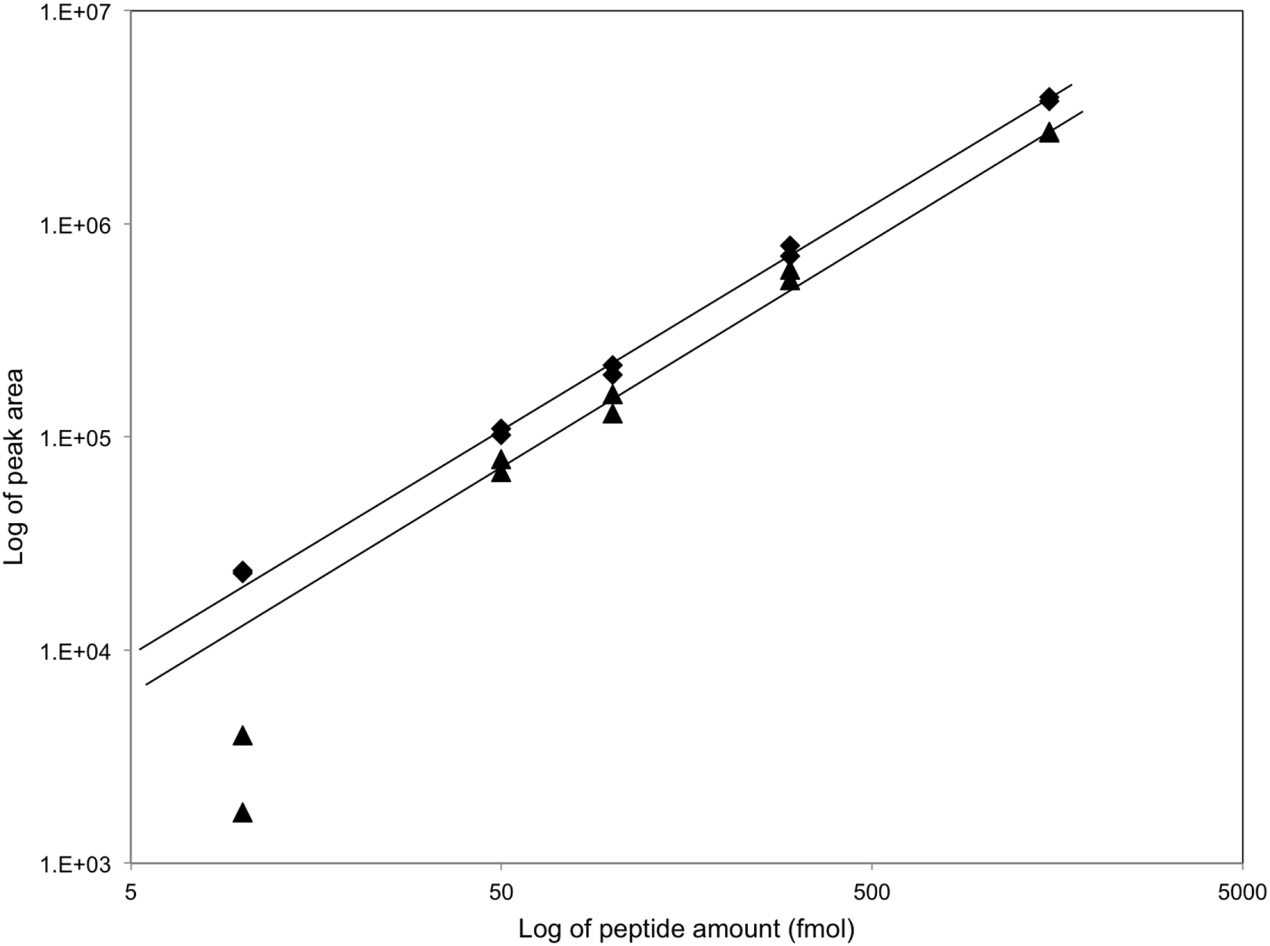
Example determinations of MRM Limit of Quantitation (LOQ) using synthetic peptides. Dilution series of peptides SLDEPSSEVVER (⧫), which is proteotypic for the *B. subtilis* GerBA protein, and EAYSDDVPEGQVVK (▴), which is proteotypic for the *B. subtilis* PrkC protein, were subjected in duplicate to MRM analysis with detection of the parent ion 493.9 m/z and the fragment ion 798.3 m/z for GerBA or the parent ion 768.7 m/z and the fragment ion 1085.6 m/z for PrkC (Table 1). Fragment ion peak areas were plotted against peptide amount, and best-fit lines were applied. LOQ’s were defined as the lowest concentration at which the response was still linear or the lowest concentration at which the contribution due to noise was such that reproducible results could be obtained (peak area ≥1×E^+4^).

Thirteen germination-related membrane proteins (Table 1) were initially targeted for MRM method development, with a list of potential proteotypic tryptic peptides generated using the Enhanced Signature Peptide Prediction tool [36] using a cutoff value of 0.6. Peptides were synthesized by JPT Peptide Technologies GmbH Inc., and were directly infused into the mass spectrometer for determination of target fragment ions and ionization conditions. For each synthesized peptide, elution times were identified, the dominant precursor ion of predicted m/z (Q1 ion) was identified and fragmented, and dominant fragment ions of expected m/z (Q3 ions) were identified and quantified. Limits of quantification (LOQ) (Table 1) were determined using the established MRM methods and dilution series from 10–1500 fmol of each synthetic peptide (Fig. 1).

**Table 1:** Peptide Details for MRM Analysis of the *B. subtilis* germination proteins.

Protein	Proteotypic peptide sequence	Parent ion	Fragment ion	LOQ
	(position in protein)	m/z (charge state)	m/z (ion)	fmoles (peak area)^a^
GerAA	LDQLDARPVETAK (77–89)	486.2 (+3)	672.0 (y_12_^+2^)	10 (3×E^+5^)
		486.2 (+3)	614.5 (y_11_^+2^)	10 (3×E^+5^)
	DEETLTLDQVK (64–74)	646.1 (+2)	703.4 (y_6_^+1^)	10 (8×E^+4^)
	VSSALFNGR (233–241)	476.3 (+2)	765.3 (y_7_^+1^)	10 (1×E^+4^)
		476.3 (+2)	374.1 (y_7_-NH_3_^+2^)	10 (1×E^+4^)
GerAC	ADVTGLGNEVR (321–331)	565.8 (+2)	744.3 (y_7_^+1^)	50 (1×E^+4^)
		565.8 (+2)	845.5 (y_8_^+1^)	100 (1×E^+4^)
		565.8 (+2)	687.4 (y_6_^+1^)	50 (1×E^+4^)
GerBA	TSDPNLVIK (156–164)	493.9 (+2)	683.2 (y_6_^+1^)	10 (2×E^+4^)
		493.9 (+2)	798.3 (y_7_^+1^)	10 (2×E^+4^)
	SLDEPSSEVVER (124–135)	674.1 (+2)	902.3 (y_8_^+1^)	10 (4×E^+4^)
		674.1 (+2)	316.2 (y_5_^+2^)	10 (3×E^+4^)
	VESSLLEGR (235–243)	495.4 (+2)	761.3 (y_7_^+1^)	10 (1×E^+4^)
GerBC	GILTEDQNPNENSFSK (279–294)	897.3 (+2)	922.4 (y_8_^+1^)	50 (1×E^+4^)
		897.3 (+2)	1036.6 (y_9_^+1^)	50 (1×E^+4^)
	GNAADVFTK (135–143)	461.8 (+2)	609.4 (y_5_^+1^)	10 (1×E^+4^)
		461.8 (+2)	680.3 (y_6_^+1^)	50 (1×E^+4^)
GerKA	ERPVLISPSLAK (31–42)	437.4 (+3)	595.3 (b_5_^+1^)	10 (2×E^+4^)
		437.4 (+3)	515.2 (y_5_^+1^)	10 (2×E^+4^)
		437.4 (+3)	602.1 (y_6_^+1^)	50 (1×E^+4^)
	SIQEPSTQVSFR (159–170)	690.2 (+2)	921.7 (y_8_^+1^)	10 (1×E^+4^)
		690.2 (+2)	329.0 (b_3_^+1^)	50 (1×E^+4^)
		690.2 (+2)	1050.5 (y_9_^+1^)	50 (1×E^+4^)
	EVGSSSDVIIR (50–60)	581.5 (+2)	789.5 (y_7_^+1^)	10 (1×E^+4^)
GerKC	TLDFTEAQYGR (166–176)	651.0 (+2)	330.2 (b_3_^+1^)	10 (1×E^+4^)
		651.0 (+2)	723.5 (y_6_^+1^)	50 (1×E^+4^)
GerD	NIFEDTDFAEGFAK (90–103)	802.5 (+2)	1376.6 (y_12_^+1^)	100 (1×E^+4^)
		802.5 (+2)	422.1 (y_4_^+1^)	300 (1×E^+4^)
		802.5 (+2)	1229.6 (y_11_^+1^)	300 (7×E^+5^)
SpoVAC	SEGLVLGVATNM(ox)FK (109–122)	741.5 (+2)	883.2 (y_8_^+1^)	50 (1×E^+4^)
		741.5 (+2)	996.3 (y_9_^+1^)	50 (1×E^+4^)
	SEGLVLGVATNMFK (109–122)	733.6 (+2)	867.3 (y_8_^+1^)	300 (1×E^+4^)
		733.6 (+2)	980.7 (y_9_^+1^)	300 (1×E^+4^)
SpoVAD	ETIPTIAHGVVFER (320–333)	523.9 (+3)	409.3 (y_11_^+3^)	50 (1×E^+4^)
		523.9 (+3)	613.6 (y_11_^+2^)	50 (1×E^+4^)
	QLMEDAVNVALQK (57–69)	730.2 (+2)	459.3 (y_4_^+1^)	100 (1×E^+4^)
		730.2 (+2)	672.3 (y_6_^+1^)	100 (1×E^+4^)
YpeB	IGVFSYVPVENK (326–337)	676.7 (+2)	586.1 (y_5_^+1^)	50 (1×E^+4^)
		676.7 (+2)	935.3 (y_8_^+1^)	50 (1×E^+4^)
	TIPKPAITEAEAK (372–384)	457.2 (+3)	577.9 (y_11_^+2^)	10 (2×E^+5^)
		457.2 (+3)	929.5 (y_9_^+1^)	10 (5×E^+4^)
	VALDDGEVVGFSAR (349–362)	718.2 (+2)	636.3 (y_6_^+1^)	100 (1×E^+5^)
		718.2 (+2)	537.4 (y_5_^+1^)	100 (3×E^+5^)
PrkC	EAASGYLEDNGLK (508–520)	684.3 (+2)	789.3 (y_7_^+1^)	10 (1×E^+4^)
		684.3 (+2)	1096.5 (y_10_^+1^)	10 (1×E^+4^)
	EAYSDDVPEGQVVK (525–538)	768.7 (+2)	756.3 (y_7_^+1^)	50 (1×E^+4^)
		768.7 (+2)	1085.6 (y_10_^+1^)	50 (1×E^+4^)
	TEIGDVTGQTVDQAK(429–443)	781.8 (+2)	947.6 (y_9_^+1^)	50 (1×E^+4^)
		781.8 (+2)	1218.8 (y_12_^+1^)	10 (1×E^+4^)

a LOQ is the limit of quantitation determined for this fragment ion, as described in Materials and Methods.

Proteins in spore membrane fractions were solubilized with 20 mM Tris-HCl [pH 8.0], 8 M urea, 45 mM dithiothreitol at a final protein concentration of 1 mg/ml, followed by a 37°C overnight trypsin digestion at 20∶1 (w/w) protein∶Trypsin ratio. The tryptic peptides were desalted and concentrated using OMIX C18 microextraction pipette tips (Varian) following the manufacturer’s protocol. Peptides was separated using an Eksigent Nano 2-D liquid chromatography system connected to a 100×0.075 mm Magic C18AQ (200 Å, 3 µm, Bruker) column packed in-house using an eFRIT fused silica capillary (Phoenix S&T). Ten microliters of each sample was first loaded onto a C18 trap cartridge at 10 µl/min for 15 minutes using solvent A (2∶98 acetonitrile∶water containing 0.1% formic acid). The trap cartridge was switched in-line with the analytical column and the trap and column were flushed with 95% solvent A, 5% solvent B (98∶2 acetonitrile∶water containing 0.1% formic acid) for 5 minutes at 300 nl/min. This was followed by a linear gradient to 86% solvent A over 5 minutes then a linear gradient to 71% solvent A over 45 minutes and finally a linear gradient to 35% solvent A over 5 minutes. The column was flushed for 2 minutes with 35% solvent A and reequilibrated at the starting conditions for 13 minutes prior to the next sample injection. The eluent was introduced into an AB Sciex 4000 QTrap mass spectrometer controlled by Analyst 1.4.2 software (AB Sciex) via a nano-electrospray source (Phoenix S&T). The mass spectrometer was operated in positive ion mode utilizing an MRM method containing precursor/product ion transitions corresponding to peptides described below. Dwell time for each transition was 40 ms and the total cycle time was 6.6 seconds. The first quadrupole was operated at low resolution while the third quadrupole was set to unit resolution. Ion spray voltage was 2400 V, curtain and sheath gases were 12 (arbitrary units), interface heater temperature was 120°C and the entrance potential was 10 V for all transitions. CAD gas was set to medium corresponding to a vacuum of 3.1×10^−5^ Torr.

### Data Collection and Refining

When determining which of the identified Q3 ion peak areas were suitable for quantitative comparisons across all samples, we applied the following raw data refining criteria. 1) The retention time of a Q3 ion in all samples should be the same as that determined for the corresponding synthetic peptide. Q3 ions that did not have consistent retention times were excluded from further analysis. 2) If a quantified peptide had less than two quantifiable Q3 ions, the peptide was excluded from further analysis. 3) If the peak area of a Q3 ion was below established limited of quantification, the Q3 ion was excluded from further analysis. 4) Among all nine samples, if the Q3 ion peaks in more than three samples had S/N ratio values less than 10, then the Q3 ion was excluded from further analysis. (The end section of each Q3 ion spectrum was considered as base line (noise) when collecting the S/N ratio for limit of quantification evaluation.).

Within each biological replicate set, there were three membrane fraction samples: dormant, germinated, and superdormant. Three biological replicates were derived from three independent spore preparations. For each quantified Q3 ion, peak area ratios between two membrane fractions were calculated only within a biological replicate set. Ratios were then compared across biological replicates. Theoretically, if a protein’s abundance was the same in two different samples, the peak area ratios for the Q3 ions of its peptides would be 1. Among all Q3 ion peak area ratios calculated, those of proteins GerAA, GerBA, and GerKA were always close to 1.0. We took these proteins to represent unchanged proteins within the samples, and pooled their Q3 ion peak area ratios to represent the level of physiological variance. For each comparison group, we then evaluated the significance of a protein change by comparing peak area ratios of the protein to this unchanged protein peak area ratio pool using a two samples student t-test. In addition, for each protein, we evaluated the significance of two comparison groups using the Student’s t-test. Both tests used two-tailed, unequal variance p values, and statistical significance for both t-tests was set at p<0.05.

## Results

### Isolation and Characterization of Spore Populations

**Figure 2:**
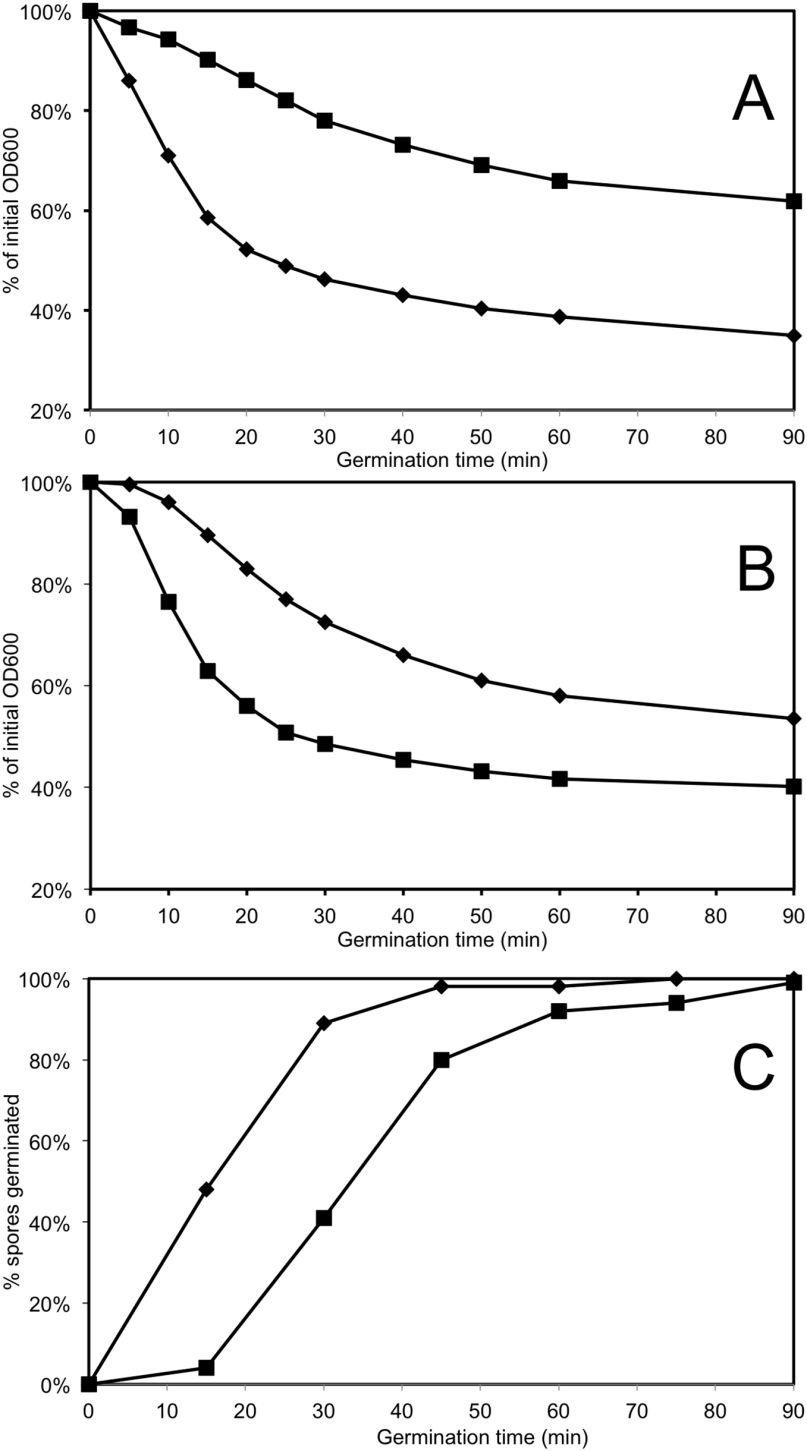
Germination of dormant and superdormant spores with nutrient and non-nutrient germinants. Superdormant spores of *B. subtilis* strain PS832 (wild type) were isolated following prolonged germination with 10 mM L-Valine as described in materials and methods. Squares (▪) indicate isolated superdormant spores, and diamonds (⧫) indicate the initial dormant spores used for isolation. A) Germination with 10 mM L- Valine; B) Germination with AGFK; C) Germination with Ca^2+^-DPA. Data from one biological replicate of dormant and superdormant spores is shown. Analyses of the other two biological replicates produced very similar results.

Three independent preparations of *B. subtilis* dormant spores were germinated using L-valine, with downstream processing producing rapidly germinating and superdormant spore populations. The yield of superdormant spores was 1.09±0.16% (n = 3); somewhat less than the 3.8% yield in a previous publication [7]. Two reported characteristics of superdormant spore populations isolated using L-valine are that the superdormant spores germinate poorly with L-valine as well as with germinants that use a different germinant receptor, with the superdormant spores being as viable as the initial dormant spores when germinated with non-nutrient germinants [7]. Our superdormant spores also germinated slowly with L-valine, in comparison with the rapid germination of the initial dormant spores (Fig. 2A). However, when using AGFK as germinant, which acts through different germinant receptors than does L-valine [12], the superdormant spores germinated more rapidly than the initial dormant spores (Fig. 2B). In addition, the superdormant spores also reached a higher efficiency of germination based on a greater OD_600_ decrease than the initial dormant spores. While our results are different from those of the original description of superdormant spores [7], similar observations were reported for superdormant spores isolated in a more recent study [8]. The effect of a non-nutrient germinant on the superdormant spores was tested using Ca^2+^-DPA, which causes activation of the GSLE CwlJ [37], bypassing part of the germination apparatus that may be deficient in superdormant spores. The superdormant spores completed Ca^2+^-DPA-triggered germination as efficiently as the initial dormant spores after an initial lag period (Fig. 2C), similar to a previous report [8]. In summary, the results of the phenotypic analyses support the claim that spores isolated after extensive L-valine germination can be classified as superdormant. To verify that these spores were not superdormant due to a genetic alteration, they were germinated and spread on plates, and 10 randomly selected colonies were selected, cultured, sporulated, and tested for germination rate. Similar to a previous report [7], spore populations produced by these strains germinated equivalently to those of the wild type strain.

### Quantification of Spore Membrane Proteins by MRM Assays

**Figure 3:**
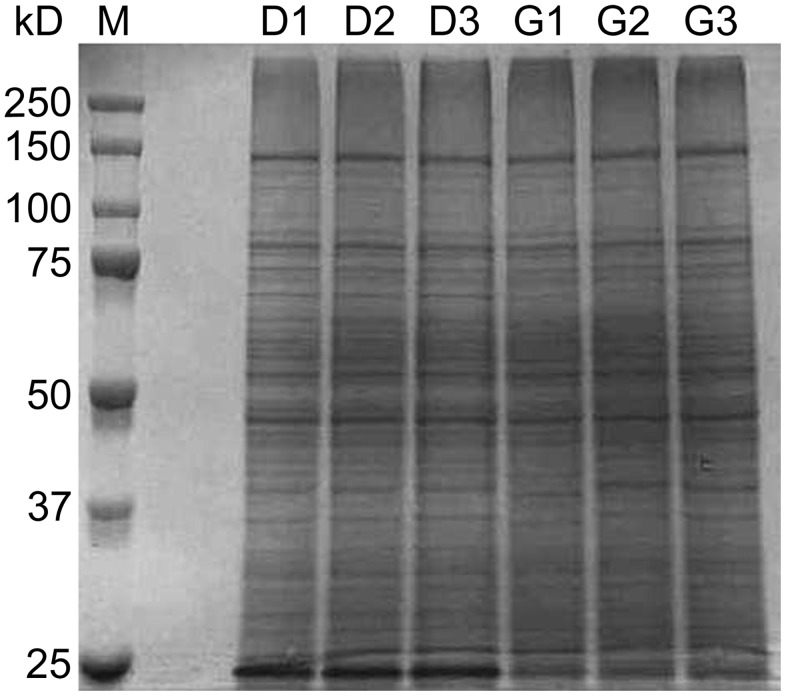
Gel electrophoresis of membrane-associated spore proteins. Membrane preparations were obtained from dormant (D) and germinated (G) spores produced from three independent spore preparations (1, 2, and 3). Protein concentrations in membrane preparations were determined by quantitative amino acid analyses, and identical protein amounts were loaded onto a 9% polyacrylamide gel. Sizes of protein standard markers (M) are indicated on the left. Proteins were stained using Coomassie blue.

Membrane samples were prepared from dormant, germinated, and superdormant spores and were used to quantify the targeted germination-related proteins relative to the total protein concentration. The total protein in each sample was determined by amino acid analysis. SDS-PAGE analysis of total proteins was consistent with this quantification and revealed essentially identical protein band patterns across biological replicates (Fig. 3).

**Figure 4:**
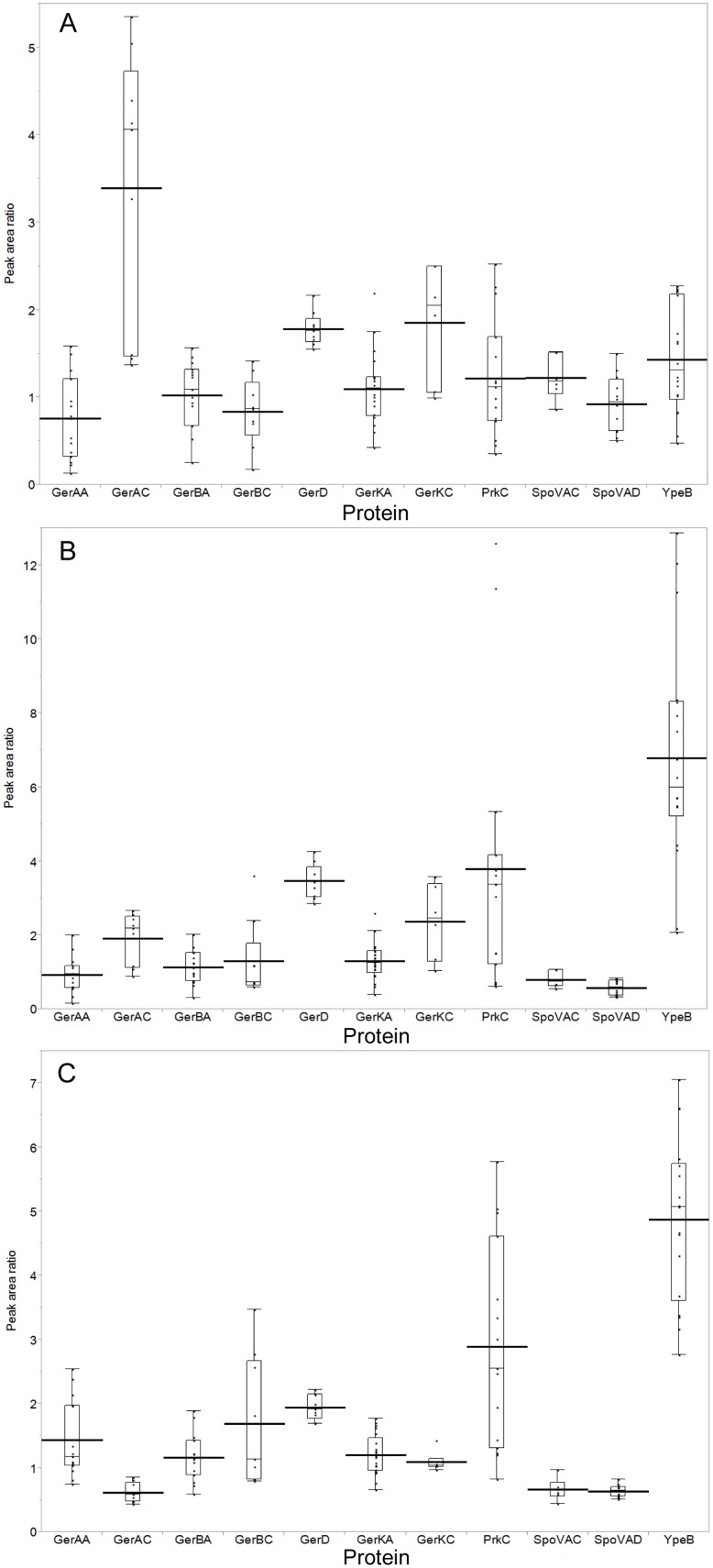
Relative quantities of germination proteins in dormant, superdormant, and germinated spores. Proteins were quantified in membrane samples by MRM analyses. The relative abundance of each protein is expressed as the ratio of each particular product ion peak area detected in dormant versus superdormant (A), dormant versus germinated (B), or superdormant versus germinated (C) spore samples derived from the same spore preparation. Ratios determined for samples from three independent spore preparations were then pooled. Each box and whisker plot indicates individual product ion ratio values (dots), 25–75 percentiles (boxes) and full ranges (whiskers), excluding statistically determined outliers. Means are indicated by the short lines traversing the boxes and medians are shown by the lines traversing the boxes.

The MRM assays centered on the detection of 11 of the 13 proteins expected to be membrane associated and involved in spore germination (Table 1). As peptides of varying compositions exhibit different ionization efficiencies, we determined LOQs for each peptide. We were not able to identify any proteotypic peptides for GerAB that were even predicted to function well in an MRM assay, and we were not able to obtain quantifiable MRM data for GerBB and GerKB due to the fact that the signal for the proteotypic peptides designed for these integral membrane proteins were below the limit of detection. Nonetheless, we were able to quantify the A and C subunits of the germinant receptors. The ratios of GerAA, GerBA, and GerKA between dormant and superdormant spores were very close to 1.0. In contrast, the amounts of GerAC and GerKC in superdormant spores were 3.4 and 1.9-fold lower than the amounts in dormant spores (Fig. 4A). These decreases of GerAC (p = 0.002) and GerKC (p = 0.023) were statistically significant. GerBC, however, showed no significant difference in amount between superdormant and dormant spores (Fig. 4A).

GerD is a lipoprotein that is localized predominantly to the spore inner membrane [32] and functions in both GerA and GerB/K-mediated germination responses [24]. GerD was 1.8-fold less abundant in membranes isolated from superdormant spores in comparison to those from dormant spores (Fig. 4A). PrkC, SpoVAC, SpoVAD, and YpeB exhibited no significant difference in abundance between superdormant and dormant spore samples (Fig. 4A).

The relative amounts of GerAA, GerBA, and GerKA in germinated spore samples were similar to the amounts of these proteins in those from dormant spores. In contrast, the amounts of GerAC, GerBC, and GerKC in germinated spore membranes decreased 1.9, 1.6, and 2.4-fold respectively in comparison to dormant spores (Fig. 4B). Previous western blot results showed that membrane-associated GerD decreased during spore germination [32]. This was confirmed in the MRM assays, which showed that membrane-associated GerD decreased 3.5-fold during spore germination (Fig. 4B). The decreases of GerAC (p = 0.009), GerKC (p = 0.032), and GerD (p<0.0001) were statistically significant.

To date, there is no report regarding PrkC function in a nutrient germinant receptor-mediated pathway. The MRM assays indicated that membrane-associated PrkC was significantly decreased 3.8-fold in amount after spore germination (Fig. 4B). Similarly, three proteotypic YpeB peptides decreased 6.8-fold in amount during spore germination (Fig. 4B). This result is consistent with a previous observation of YpeB degradation during germination [23]. A fourth YpeB peptide was clearly quantifiable in dormant spore samples, but was undetectable in germinated spore membrane fractions. This peptide is near the YpeB N-terminus (residues 57–67), whereas the three detectable YpeB peptides were closer to the C terminus.

SpoVAC and SpoVAD proteins were chosen as representatives of the *spoVA*-encoded proteins. SpoVAC is predicted to be an integral membrane protein [18], [38], and SpoVAD is more likely to be a peripheral membrane protein based on its crystal structure [39]. While SpoVAD could be detected in both dormant and germinated membrane fraction samples by Western-blot (data not shown), the MRM assays indicated that SpoVAC and SpoVAD significantly increased 1.3 and 1.7-fold respectively in germinated spore membrane samples in comparison to those of dormant spores (Fig. 4B).

The germinant receptor A subunit amounts detected were similar in superdormant and germinated spore samples. While membranes of both superdormant and germinated spores had significantly less GerAC and GerKC proteins relative to dormant spores, the decrease in GerAC was significantly greater than that of GerKC and was 1.6-fold lower in superdormant spores in comparison to germinated spores (Fig. 4C). Similarly, while GerD was also less abundant in membranes from both superdormant and germinated spores than in dormant spores, the difference was greater in the germinated spores, such that GerD was 2-fold more abundant in superdormant spores than in germinated spores (Fig. 4C). This difference was statistically significant (p<0.0001). If germination-induced protein changes initiated but did not progress past GerD in superdormant spores, the difference in abundance for proteins involved in later germination events in comparison to germinated spores should be similar to a dormant/germinated spores comparison. Indeed, our results showed that YpeB and PrkC were 4.9 and 2.9-fold more abundant in samples from superdormant spores than in those from germinated spores, and SpoVAC and SpoVAD were 1.6 and 1.5-fold less abundant in superdormant spore samples than in germinated spore samples (Fig. 4C).

## Discussion

Spore germination starts at the spore inner membrane with the interaction of germinant with Ger receptor proteins and progresses through core rehydration and cortex breakdown. Deficiencies in Ger receptors and associated proteins, Ca^2+^-DPA channels, and lytic enzymes can potentially inhibit the germination process, leading to the production of superdormant spores. In this work, L-valine superdormant spores responded poorly to valine but germinated well with AGFK; a result that is different from the initial report of superdormant spores [7] but consistent with a later report [8]. In contrast to a high yield (12%) of superdormant spores isolated with AGFK in previous work [7], we were unable to isolate any superdormant spores using AGFK. The reasons for these differences are not clear, but raise the possibility that there may be multiple pathways to superdormancy, and slight differences in the method of preparation and isolation may result in significantly different superdormant spore populations.

Our quantitative MRM assays were performed on membrane samples derived from broken spores. Results published while this work was in progress indicate that these samples may represent a subset of the spore membrane fraction, as extensive chemical extraction is required to recover the full amount of several spore membrane proteins [40]. While the sample analyzed here may not represent the entire spore membrane fraction, two lines of evidence indicate that the samples recovered from each spore type are similar fractions. The relative abundance of the majority of the proteins analyzed was the same in dormant and superdormant spores, and the amounts of the integral membrane A subunits of the Ger receptors were not altered in germinated spores, indicating that the membrane fractions obtained from the different spore types were comparable in their protein complements.

We interpret the similarity in Ger receptor A protein abundance in dormant and superdormant spore fractions to imply that the isolated superdormant population is not delayed in germination due to a low level of Ger receptors. These results differ from a previous publication that reported GerAA and GerAC protein levels were 7-fold lower and other GR subunits 3-fold lower in superdormant spores [28]. We did find that the C subunits of the GerA and GerK receptors were decreased in samples from our superdormant spores. Germination-induced decreases in C subunit abundance, relative to dormant spores, were in one case less (GerAC) and in other cases slightly greater (GerBC and GerKC) than those observed in superdormant spores. As the C subunits are believed to be present in a stoichiometric association with the A subunits in the membranes of dormant spores [40], a decrease in the C subunits may indicate a change that takes place during early germination or may indicate a change in the maintenance of subunit association with the membrane during membrane fraction washing and isolation. Published data indicate that GerD, a lipoprotein like the C subunits, can be partially extracted by a high salt wash similar to that used in this study and is released from the membrane during germination [32]. The decreased abundance of C subunits observed in superdormant spore samples could be either a cause of superdormancy or a result of the superdormant spore isolation process. Protein associations could decrease during Ger receptor activation upon germinant binding, and the membrane-association may be less stable as the spore membrane regains fluidity during germination [41]. Prolonged exposure to germinants during the superdormant spore isolation may result in the loss of C subunits from the membrane, with a blockage in the germination pathway at a point downstream of the Ger receptors.

An absence of GerD was previously shown to result in a germination initiation defect [24], [27]. Previous studies also indicated that the germination receptors and GerD co-localize to a discrete cluster on the membrane [42] and that SpoVA proteins can associate with Ger receptors [43], and thus we expect that the comparable membrane factions we derived from different spore types would contain similar amounts of GerD. Superdormant spore membrane samples had 2-fold less GerD than those from dormant spores, and germinated spore samples contained even less GerD. Similar to the case for the Ger receptor C subunits, the decreased level of GerD in superdormant spores could be either a cause of superdormancy or a result of a partial germination response that is blocked at a subsequent point.

Other germination-active proteins, PrkC, SpoVAC, SpoVAD, and YpeB were present in dormant and superdormant spore membrane preparations in similar quantities, but did show changes in abundance during germination. This suggests that any early germination-related events that have taken place within the superdormant spores did not progress to later events such as SpoVA-assisted DPA release [17] or YpeB-related cortex degradation [23]. Interestingly, the abundance of membrane-associated SpoVAC and SpoVAD proteins increased during germination. As this increase is taking place well before any new protein synthesis, it must represent an increased association of the proteins with the membrane or with other membrane-bound proteins, most likely other products of the SpoVA DPA-transport complex. Several studies indicate that SpoVAD localizes to the spore inner membrane [44], [45], and recent structural analyses of this protein show that it is likely to be a peripheral membrane protein [39], [46]. In our western-blot analysis of SpoVAD, it was detected in both soluble and membrane fractions, suggesting a weak membrane association (data not shown). Another novel finding is that PrkC abundance decreased significantly during spore germination triggered by L-Val. Although this protein was previously demonstrated to be a germinant receptor that responds to muropeptides [19], there is no report of its activity in other germinant receptor-mediated pathways.

The most dramatic change in protein abundance during germination was that of YpeB, which is required for incorporation of SleB in spore and thus for normal cortex degradation during germination [23]. This result is consistent with a previous observation of YpeB degradation during germination. The 52 kDa YpeB is processed to a ∼30 kDa product during germination [23]. The 6.8-fold decrease in YpeB abundance we observed was calculated using those peptides we could detect in both dormant and germinated spore samples, all of which were in the C-terminal half of the protein. One peptide nearer the N-terminus of YpeB was detected in dormant spore samples but was undetectable in germinated spore samples, indicating a decrease of >14-fold. This differential loss of peptides indicates that the more stable 30-kDa portion of YpeB represents a C-terminal portion. Ongoing studies in our lab are consistent with this (data not shown). It is not clear if the 6.8-fold decrease in the observed YpeB peptides is due to protein degradation, a decrease in membrane association, or both. Because the N-terminus of YpeB apparently contains an uncleaved signal peptide, proteolytic removal of this domain would be expected to decrease YpeB-membrane association.

Studies of spore germination heterogeneity have shown that the major variable in kinetics of nutrient-triggered spore germination is the germination initiation time, termed T_lag_, with the range of superdormant spores’ T_lag_ times being significantly greater than that of dormant spores [8]. Previous studies also showed that *gerD* spores had significantly longer T_lag_ times than wild-type spores [27]. The results from our work in relation to those previous efforts lead us to propose that decreased abundance of GerD can be a contributing factor in superdormancy.

## References

[1] Leggett MJ, McDonnell G, Denyer SP, Setlow P, Maillard JY (2012) Bacterial spore structures and their protective role in biocide resistance. J Appl Microbiol 113: 485–498. 22574673 10.1111/j.1365-2672.2012.05336.x

[2] Riesenman PJ, Nicholson WL (2000) Role of the spore coat layers in *Bacillus subtilis* spore resistance to hydrogen peroxide, artificial UV-C, UV-B, and solar UV radiation. Appl Environ Microbiol 66: 620–626. 10653726 10.1128/aem.66.2.620-626.2000 PMC91871

[3] Setlow P (2003) Spore germination. Curr Opin Microbiol 6: 550–556. 14662349 10.1016/j.mib.2003.10.001

[4] Mallozzi M, Viswanathan VK, Vedantam G (2010) Spore-forming Bacilli and Clostridia in human disease. Future Microbiol 5: 1109–1123. 20632809 10.2217/fmb.10.60

[5] Kong L, Zhang P, Setlow P, Li YQ (2010) Characterization of bacterial spore germination using integrated phase contrast microscopy, Raman spectroscopy, and optical tweezers. Anal Chem 82: 3840–3847. 20369827 10.1021/ac1003322

[6] Wang G, Zhang P, Paredes-Sabja D, Green C, Setlow P, et al (2011) Analysis of the germination of individual *Clostridium perfringens* spores and its heterogeneity. J Appl Microbiol 111: 1212–1223. 21883730 10.1111/j.1365-2672.2011.05135.x

[7] Ghosh S, Setlow P (2009) Isolation and characterization of superdormant spores of *Bacillus* species. J Bacteriol 191: 1787–1797. 19136594 10.1128/JB.01668-08 PMC2648361

[8] Zhang P, Kong L, Wang G, Scotland M, Ghosh S, et al (2012) Analysis of the slow germination of multiple individual superdormant *Bacillus subtilis* spores using multifocus Raman microspectroscopy and differential interference contrast microscopy. J Appl Microbiol 112: 526–536. 22212253 10.1111/j.1365-2672.2011.05230.x

[9] Moir A (2006) How do spores germinate? J Appl Microbiol 101: 526–530. 16907803 10.1111/j.1365-2672.2006.02885.x

[10] Corfe BM, Sammons RL, Smith DA, Mauel C (1994) The *gerB* region of the *Bacillus subtilis* 168 chromosome encodes a homologue of the *gerA* spore germination operon. Microbiology 140 (Pt 3): 471–478. 10.1099/00221287-140-3-471 8012571

[11] Zuberi AR, Feavers IM, Moir A (1985) Identification of three complementation units in the *gerA* spore germination locus of *Bacillus subtilis* . J Bacteriol 162: 756–762. 2985546 10.1128/jb.162.2.756-762.1985 PMC218915

[12] Moir A, Kemp EH, Robinson C, Corfe BM (1994) The genetic analysis of bacterial spore germination. J Appl Bacteriol 77: 9S–16S. 7989261

[13] Ramirez-Peralta A, Gupta S, Butzin XY, Setlow B, Korza G, et al (2013) Identification of new proteins that modulate the germination of spores of *Bacillus* species. J Bacteriol 195: 3009–3021. 23625846 10.1128/JB.00257-13 PMC3697528

[14] Igarashi T, Setlow B, Paidhungat M, Setlow P (2004) Effects of a *gerF* (*lgt*) mutation on the germination of spores of *Bacillus subtilis* . J Bacteriol 186: 2984–2991. 15126458 10.1128/JB.186.10.2984-2991.2004 PMC400631

[15] Atluri S, Ragkousi K, Cortezzo DE, Setlow P (2006) Cooperativity between different nutrient receptors in germination of spores of *Bacillus subtilis* and reduction of this cooperativity by alterations in the GerB receptor. J Bacteriol 188: 28–36. 16352818 10.1128/JB.188.1.28-36.2006 PMC1317597

[16] Tovar-Rojo F, Chander M, Setlow B, Setlow P (2002) The products of the *spoVA* operon are involved in dipicolinic acid uptake into developing spores of *Bacillus subtilis* . J Bacteriol 184: 584–587. 11751839 10.1128/JB.184.2.584-587.2002 PMC139579

[17] Vepachedu VR, Setlow P (2007) Role of SpoVA proteins in release of dipicolinic acid during germination of *Bacillus subtilis* spores triggered by dodecylamine or lysozyme. J Bacteriol 189: 1565–1572. 17158659 10.1128/JB.01613-06 PMC1855772

[18] Fort P, Errington J (1985) Nucleotide sequence and complementation analysis of a polycistronic sporulation operon, *spoVA*, in *Bacillus subtilis* . J Gen Microbiol 131: 1091–1105. 3926949 10.1099/00221287-131-5-1091

[19] Shah IM, Laaberki MH, Popham DL, Dworkin J (2008) A eukaryotic-like Ser/Thr kinase signals bacteria to exit dormancy in response to peptidoglycan fragments. Cell 135: 486–496. 18984160 10.1016/j.cell.2008.08.039 PMC2892110

[20] Popham DL, Helin J, Costello CE, Setlow P (1996) Muramic lactam in peptidoglycan of *Bacillus subtilis* spores is required for spore outgrowth but not for spore dehydration or heat resistance. Proc Natl Acad Sci U S A 93: 15405–15410. 8986824 10.1073/pnas.93.26.15405 PMC26417

[21] Boland FM, Atrih A, Chirakkal H, Foster SJ, Moir A (2000) Complete spore-cortex hydrolysis during germination of *Bacillus subtilis* 168 requires SleB and YpeB. Microbiology 146 (Pt 1): 57–64. 10.1099/00221287-146-1-57 10658652

[22] Ishikawa S, Yamane K, Sekiguchi J (1998) Regulation and characterization of a newly deduced cell wall hydrolase gene (*cwlJ*) which affects germination of *Bacillus subtilis* spores. J Bacteriol 180: 1375–1380. 9515903 10.1128/jb.180.6.1375-1380.1998 PMC107033

[23] Chirakkal H, O’Rourke M, Atrih A, Foster SJ, Moir A (2002) Analysis of spore cortex lytic enzymes and related proteins in *Bacillus subtilis* endospore germination. Microbiology 148: 2383–2392. 12177332 10.1099/00221287-148-8-2383

[24] Pelczar PL, Igarashi T, Setlow B, Setlow P (2007) Role of GerD in germination of *Bacillus subtilis* spores. J Bacteriol 189: 1090–1098. 17122337 10.1128/JB.01606-06 PMC1797312

[25] Stewart KA, Yi X, Ghosh S, Setlow P (2012) Germination Protein Levels and Rates of Germination of Spores of *Bacillus subtilis* with Overexpressed or Deleted Genes Encoding Germination Proteins. J Bacteriol 194: 3156–3164. 22493018 10.1128/JB.00405-12 PMC3370836

[26] Cabrera-Martinez RM, Tovar-Rojo F, Vepachedu VR, Setlow P (2003) Effects of overexpression of nutrient receptors on germination of spores of *Bacillus subtilis* . J Bacteriol 185: 2457–2464. 12670969 10.1128/JB.185.8.2457-2464.2003 PMC152624

[27] Wang G, Yi X, Li YQ, Setlow P (2011) Germination of individual *Bacillus subtilis* spores with alterations in the GerD and SpoVA proteins, which are important in spore germination. J Bacteriol 193: 2301–2311. 21398556 10.1128/JB.00122-11 PMC3133087

[28] Ghosh S, Scotland M, Setlow P (2012) Levels of germination proteins in dormant and superdormant spores of *Bacillus subtilis* . J Bacteriol 194: 2221–2227. 22343299 10.1128/JB.00151-12 PMC3347068

[29] Picotti P, Rinner O, Stallmach R, Dautel F, Farrah T, et al (2010) High-throughput generation of selected reaction-monitoring assays for proteins and proteomes. Nat Methods 7: 43–46. 19966807 10.1038/nmeth.1408

[30] Leighton TJ, Doi RH (1971) The stability of messenger ribonucleic acid during sporulation in *Bacillus subtilis* . J Biol Chem 254: 3189–3195. 4995746

[31] Nicholson WL, and P Setlow. (1990) Sporulation, germination, and outgrowth. Molecular methods for bacillus. Chichester, England: John Wiley & Sons Ltd. P. 391–450.

[32] Pelczar PL, Setlow P (2008) Localization of the germination protein GerD to the inner membrane in *Bacillus subtilis* spores. J Bacteriol 190: 5635–5641. 18556788 10.1128/JB.00670-08 PMC2519376

[33] Hahne H, Wolff S, Hecker M, Becher D (2008) From complementarity to comprehensiveness–targeting the membrane proteome of growing *Bacillus subtilis* by divergent approaches. Proteomics 8: 4123–4136. 18763711 10.1002/pmic.200800258

[34] Paidhungat M, Setlow P (2001) Localization of a germinant receptor protein (GerBA) to the inner membrane of *Bacillus subtilis* spores. J Bacteriol 183: 3982–3990. 11395462 10.1128/JB.183.13.3982-3990.2001 PMC95281

[35] González-Castro MJ, López-Hernández J, Simal-Lozano J, Oruña-Concha MJ (1997) Determination of amino acids in green beans by derivitization with phenylisothiocyanate and high-performance liquid chromatography with ultraviolet detection. J Chrom Sci 35: 181–185.

[36] Fusaro VA, Mani DR, Mesirov JP, Carr SA (2009) Prediction of high-responding peptides for targeted protein assays by mass spectrometry. Nat Biotechnol 27: 190–198. 19169245 10.1038/nbt.1524 PMC2753399

[37] Paidhungat M, Ragkousi K, Setlow P (2001) Genetic requirements for induction of germination of spores of *Bacillus subtilis* by Ca(2+)-dipicolinate. J Bacteriol 183: 4886–4893. 11466292 10.1128/JB.183.16.4886-4893.2001 PMC99543

[38] Paredes-Sabja D, Setlow B, Setlow P, Sarker MR (2008) Characterization of *Clostridium perfringens* spores that lack SpoVA proteins and dipicolinic acid. J Bacteriol 190: 4648–4659. 18469104 10.1128/JB.00325-08 PMC2446781

[39] Forouhar F, Su M, Seetharaman J, Fang F, Xiao R, et al. (2010) Crystal structure of Stage V sporulation protein AD (SpoVAD) from *Bacillus subtilis*, Northeast Structural Genomics Consortium Target SR525. PDB id: 3LM6.

[40] Stewart KA, Setlow P (2013) Numbers of individual nutrient germinant receptors and other germination proteins in spores of *Bacillus subtilis* . J Bacteriol 195: 3575–3582. 23749970 10.1128/JB.00377-13 PMC3754565

[41] Cowan AE, Olivastro EM, Koppel DE, Loshon CA, Setlow B, et al (2004) Lipids in the inner membrane of dormant spores of *Bacillus* species are largely immobile. Proc Natl Acad Sci USA 101: 7733–7738. 15126669 10.1073/pnas.0306859101 PMC419675

[42] Griffiths KK, Zhang J, Cowan AE, Yu J, Setlow P (2011) Germination proteins in the inner membrane of dormant *Bacillus subtilis* spores colocalize in a discrete cluster. Mol Microbiol 81: 1061–1077. 21696470 10.1111/j.1365-2958.2011.07753.x PMC7959159

[43] Vepachedu VR, Setlow P (2007) Analysis of interactions between nutrient germinant receptors and SpoVA proteins of *Bacillus subtilis* spores. FEMS Microbiol Lett 274: 42–47. 17573930 10.1111/j.1574-6968.2007.00807.x

[44] Korza G, Setlow P (2013) Topology and accessibility of germination proteins in the *Bacillus subtilis* spore inner membrane. J Bacteriol 195: 1484–1491. 23335419 10.1128/JB.02262-12 PMC3624538

[45] Vepachedu VR, Setlow P (2005) Localization of SpoVAD to the inner membrane of spores of *Bacillus subtilis* . J Bacteriol 187: 5677–5682. 16077113 10.1128/JB.187.16.5677-5682.2005 PMC1196082

[46] Li Y, Davis A, Korza G, Zhang P, Li YQ, et al (2012) Role of a SpoVA protein in dipicolinic acid uptake into developing spores of *Bacillus subtilis* . J Bacteriol 194: 1875–1884. 22328679 10.1128/JB.00062-12 PMC3318455

